# Cysticerci in the Brain Diagnosticated during Life

**Published:** 1879-02

**Authors:** 


CYSTICERCI IN THE BRAIN DIAGNOSTICATED DURING LIFE. A case of this character is recorded by Dr. Joseph Pollak in the *Wiener Med. Presse*, No. 47, 1878. The patient was a boy eight years of age. Examination of the pulse, temperature, thoracic and abdominal viscera failed to reveal anything abnormal. The boy complained of excruciating headache, and his piercing cries were loud enough to be heard at quite a distance. Very shortly after his first visit the attendant was recalled, when he found the pupils dilated, the urine and fæces passed involuntarily, the abdomen distended; headache was still severe. Every few hours, attacks of an epileptiform nature recurred, while in the intervals there was a remarkable absence of all these symptoms. At one of his visits just after prescribing a cathartic, he had occasion to examine the stools, where he found portions of a tænia. The presence of this, in connection with the other symptoms, at once aroused the suspicion that he had here a case of entozoal origin. At his next visit he found the patient comatose, and on examination of his pupils found, to his surprise, what proved on a closer examination, to be a cysticercus in the anterior chamber. He at once pronounced the case one of cysticercus of the brain. The patient died shortly afterward, and the diagnosis was fully verified.

